# Brilacidin as a Broad-Spectrum Inhibitor of Enveloped, Acutely Infectious Viruses

**DOI:** 10.3390/microorganisms12010054

**Published:** 2023-12-28

**Authors:** Carol A. Anderson, Michael D. Barrera, Niloufar A. Boghdeh, Miata Smith, Farhang Alem, Aarthi Narayanan

**Affiliations:** Center for Infectious Disease Research, School of Systems Biology, George Mason University, Manassas, VA 20110, USA; cander47@gmu.edu (C.A.A.); mbarrer@gmu.edu (M.D.B.); nboghdeh@gmu.edu (N.A.B.); msmit58@gmu.edu (M.S.); falem@gmu.edu (F.A.)

**Keywords:** alphavirus, bunyavirus, Echovirus, therapeutic, small molecule, viral envelope

## Abstract

Alphaviruses, belonging to the *Togaviridae* family, and bunyaviruses, belonging to the *Paramyxoviridae* family, are globally distributed and lack FDA-approved vaccines and therapeutics. The alphaviruses Venezuelan equine encephalitis virus (VEEV) and eastern equine encephalitis virus (EEEV) are known to cause severe encephalitis, whereas Sindbis virus (SINV) causes arthralgia potentially persisting for years after initial infection. The bunyavirus Rift Valley Fever virus (RVFV) can lead to blindness, liver failure, and hemorrhagic fever. Brilacidin, a small molecule that was designed de novo based on naturally occurring host defensins, was investigated for its antiviral activity against these viruses in human small airway epithelial cells (HSAECs) and African green monkey kidney cells (Veros). This testing was further expanded into a non-enveloped Echovirus, a *Picornavirus*, to further demonstrate brilacidin’s effect on early steps of the viral infectious cycle that leads to inhibition of viral load. Brilacidin demonstrated antiviral activity against alphaviruses VEEV TC-83, VEEV TrD, SINV, EEEV, and bunyavirus RVFV. The inhibitory potential of brilacidin against the viruses tested in this study was dependent on the dosing strategy which necessitated compound addition pre- and post-infection, with addition only at the post-infection stage not eliciting a robust inhibitory response. The inhibitory activity of brilacidin was only modest in the context of the non-enveloped *Picornavirus* Echovirus, suggesting brilacidin may be less potent against non-enveloped viruses.

## 1. Introduction

Alphaviruses are a diverse group of enveloped RNA viruses that can cause infection in both humans and livestock [1]. They have a single-stranded positive-sense RNA genome of about 11.5 kb in length. The genome translates into a polyprotein that is then cleaved into four nonstructural proteins (nsP1–nsP4), a capsid protein (Cp), and envelope proteins (E1 and E2). E1 and E2 generate the viral spike protein mediating viral entry through endocytosis. Interactions between Cp-E2 and the formation of the E1/E2 heterodimer drive the budding process at the host cell surface [1].

The alphaviruses Venezuelan equine encephalitis virus (VEEV) and Eastern equine encephalitis virus (EEEV) are spread to humans through mosquitos and can cause symptoms such as fever, rash, joint pain, inflammation, and malaise [2]. VEEV and EEEV tend to be encephalitogenic in nature and are endemic to South and North America, respectively. They can cause severe damage to the central nervous system, including seizures, blindness, permanent neurological damage, and death [3]. Both VEEV and EEEV are of concern due to their potential use as biological weapons and the possibility of re-emergence. There are currently no vaccines or therapeutics available for either of these viruses, which are classified as Category B priority pathogens by the National Institute of Allergy and Infectious Diseases (NIAID) and Select Agents by the Center for Disease Control (CDC) and United States Department of Agriculture (USDA).

Sindbis virus (SINV), another alphavirus, is widely distributed across Africa, Asia, Europe, and Australia [4]. SINV typically manifests as flu-like symptoms, including rash, nausea, and arthralgia that can persist for years after initial infection [5,6]. A considerable number of infected patients are left with persistent joint pain for years and, in some cases, chronic arthritis. The potential for long-term effects increases in high-risk populations, such as women between the ages of 45 and 65 [7]. Currently, there are no vaccines or therapeutics available for the treatment of SINV.

Rift Valley Fever virus (RVFV), the causative agent of Rift Valley Fever, belonging to the bunyavirus family, is a zoonotic, negative-stranded, enveloped RNA virus. Its capsid contains a tripartite genome: small (S), medium (M), and large (L), coding for the viral polymerase, two non-structural proteins (NS), a nucleoprotein (N), and two glycoproteins (Gn and Gc). Virulence is increased by the non-structural proteins, which aid in hijacking host transcription and degradation of dsRNA-dependent protein kinase, and also reduce the production of interferons in response to viral infection [8]. In livestock, RVFV causes severe disease, including hemorrhagic fever, febrile illness, fetal malformation, and spontaneous abortions. It is primarily transmitted to humans through *Aedes* and *Culex* mosquitoes. In humans, RVFV is fatal in 1–2% of cases with symptoms, including blindness, hemorrhagic fever, and miscarriages [9].

RVFV was first detected in Kenya in 1931 and since then has spread throughout Africa, Madagascar, and the Arabian Peninsula. Although not detected in the Americas, Asia, or Europe as of this writing, the emergence of other diseases such as West Nile virus (WNV) and Zika virus serves as a warning of RVFV’s potential spread. RVFV is classified as a Category A pathogen by NIAID [10].

An Echovirus (EV), belonging to the *Picornaviridae* family, was also investigated in this study. Like VEEV and EEEV, EV is a positive-sense RNA virus but is non-enveloped. The genome codes for four primary proteins are VP1-VP4. Translation results in a polyprotein that is further cleaved to form the icosahedral shell and the interior lining of the fully formed virion. VP2 and VP3 are further cleaved into replication proteins to synthesize new RNA. Fully formed virions escape the host cell by lysis [11]. This family of viruses ranges in infection severity, from asymptomatic cases to those that can result in severe central nervous system disease and meningitis [12].

Antimicrobial peptides (AMPs) constitute an interesting strategy for the development of new countermeasure strategies against such acutely infectious viruses in addition to small molecules. Recent studies show both bioactive and synthetically developed small peptides possess antiviral activities, positioning them as promising therapeutic candidates [13,14]. Brilacidin (PMX-30063) is a small molecule designed to mimic the properties of naturally occurring human defensins. Defensins are a common family of AMPs found in high concentrations in the granules of leukocytes, such as neutrophils and epithelial cells [15]. Their amphipathic nature enables them to target and disrupt lipid membranes, acting as a first line of defense against various pathogens, including viruses [16]. The development of natural defensins for therapeutic use has been limited due to toxicity, malabsorption, and high cost for production [17]. Brilacidin is smaller than a conventional defensin, cost-efficient to produce, and has been tailored to exhibit increased potency and in vivo stability based on desirable physicochemical characteristics.

Brilacidin’s ability to impact the integrity of viruses such that inhibition may be achieved has previously been shown for various coronaviruses, including SARS-CoV-2 [18,19]. The current manuscript delivers early proof-of-concept data for the potential application of brilacidin as a countermeasure strategy against enveloped, vector-transmitted viruses using alphaviruses and bunyaviruses as prototype pathogens. The inhibitory activity of brilacidin on a non-enveloped Echovirus is included for comparison to evaluate the relative efficacy of brilacidin in the context of enveloped versus non-enveloped viruses.

## 2. Materials and Methods

### 2.1. Cell Culture

Vero African green monkey kidney cells (ATCC, CCL-81, Manassas, VA, USA) were cultured with Dulbecco’s Modified Eagle’s Medium (DMEM, Quality Biological, 112-013,101CS, Gaithersburg, MD, USA) supplemented with 5% heat-inactivated fetal bovine serum (FBS), 1% penicillin and streptomycin antibiotics (Corning 30-003-CI, Corning, NY, USA), and 1% L-glutamine (Corning, 25-005-CI, Corning, NY, USA). Human small airway epithelial cells (HSAECs) were cultured with Ham’s F-12 GlutaMAX Supplement (ThermoFisher Scientific, 31765-035, Burlington, ON, Canada), 5% FBS, 1% L-glutamine, 1% penicillin and streptomycin, 1% non-essential amino acid solution (ThermoFisher Scientific, 11140-050, Grand Island, NY, USA), 1% sodium pyruvate (VWR, 45000-710, VA, USA) 0.01% 2-Mercaptoethanol (VWR Life Science, 76177-742, Radnor, PA, USA). All cells were incubated at 37 °C with 5% CO_2_ supplementation.

### 2.2. Viral Stocks

RVFV (Rift Valley Fever virus) recombinant (r)MP12 attenuated strain was derived from ZH501 strain by 12 serial passages in MRC5 cells and grown in HSAECs utilized under BSL-2 conditions [20]. The RVFV virulent strain ZH501 was obtained from Stuart Nichol, Centers for Disease Control and Prevention (CDC), and utilized under BSL-3 conditions. The full virulent Trinidad donkey (TrD) strain of VEEV (Venezuelan Equine Encephalitis Virus) was used to conduct BSL-3 studies, whereas the TC-83 strain was studied as the BSL-2 model. VEEV TC-83 and VEEV TrD were obtained from BEI Resources in Manassas, VA. TC-83 is a live attenuated vaccine derivative of the TrD strain of VEEV derived by 83 serial passages of the virus in guinea pig heart cells [21] and expanded in Vero E6 cells and quantitated using plaque assay. A VEEV TC-83 V5 E2 tagged virus was utilized as described in Barrera et al. [22]. Wild-type Eastern Equine Encephalitis Virus (EEEV) GA97 was obtained from Dr. Jonathan Jacobs (MRIGlobal) and expanded in Vero cells and quantitated via plaque assay. Sindbis virus (SINV, EgAr339) was utilized under BSL-2 conditions and was obtained from BEI Resources in Manassas, VA. Human Echovirus 6 (EV) was obtained from American Type Culture Collection (ATCC, VR-1045, Manassas, VA, USA). All select agents used in the manuscript are registered under the Center for Infectious Disease Research (CIDR, formerly NCBID) and conducted at George Mason University’s Biomedical Research Laboratory with registrations in accordance with Federal Select Agent regulations.

### 2.3. Inhibitor

Brilacidin was provided by Innovation Pharmaceuticals Inc. (Wakefield, MA, USA) and was suspended in dimethyl sulfoxide (DMSO).

### 2.4. Viral Infections

Vero cells were seeded in 96-well plates at a seeding density of 5 × 10^4^ cells per well and allowed to reach 90–100% confluency for 24 h. These cells were used for VEEV-TC-83 and TrD, EEEV, and SINV infections. HSAECs were seeded in 96-well plates at a seeding density of 5 × 10^4^ cells per well and were used for RVFV infections. Brilacidin was dissolved in dimethyl sulfoxide (DMSO). Four treatment strategies were used. Cells were pre-treated with brilacidin at 20 µM for 1 h. Pre-treatment was removed and replaced with viral inoculum for 1 hr. All cells were infected with a multiplicity of infections (MOI 0.1). Viral inoculum was removed and replaced with fresh media and 20 µM of brilacidin until collection time. For VEEV TC-83, VEEV TrD, EEEV GA97, and SINV EgAr338 infections, collection time was 18 h post-infection (hpi). For RVFV MP-12 and RVFV infections, collection time was 16 h post-infection. EV infections were collected 24 h post-infection. For direct viral treatment, virus was suspended in appropriate cell media with 20 µM of brilacidin and incubated for 1 h before being placed on cells for 1 h. The inoculum was then replaced with fresh culture media until collection time. A combination of these treatment strategies was also used. Post-treatment consisted of infecting the cells with virus for 1 h and then placing 20 µM of brilacidin on cells until collection time. Mock-infected wells consisted of treated and untreated cells during infections. Samples were collected and used immediately or frozen at –80 °C.

For IC50 infections, Vero cells were seeded in a 96-well plate at a seeding density of 1 × 10^4^ cells per well. Cells were pre-treated with varying concentrations of brilacidin for 1 h, with the upper limit being 200 μM and conducting 2-fold serial dilutions to establish an 8-point curve. The lower limit was 1.56 μM. Simultaneously, VEEV TC-83 or RVFV MP-12 were suspended in Vero media along concentration curve for 1 h. Pre-treatment was removed and cells were infected. Inoculum was removed 1 h later and cells were post-treated following the same guidance as outlined in the pre-treatment. Samples were collected 18 hpi for VEEV TC-84 and 16 hpi for RVFV MP-12.

### 2.5. Cell Viability and Toxicity Screens

Vero cells and HSAECs were seeded in 96-well white plates at 5 × 10^4^ cells per well, respectively, and allowed to grow for 24 h. Brilacidin was diluted to the desired concentrations in appropriate cell culture media. Dilutions of brilacidin were applied to individual wells of the plate and incubated at 37 °C with 5% CO_2_ for 24 h. Brilacidin and media mixture were removed, and cell viability was measured with CellTiter-Glo^®^ Luminescent Cell Viability Assay per manufacturer’s instructions (Promega, G7572, Madison, WI, USA). Luminescence was measured using GloMax Explorer Plate Reader (Promega, GM3510, Madison, WI, USA).

Viral infections were conducted as previously outlined in 96-well white plates. At specified collection time, CellTiter-Glo^®^ Luminescent Cell Viability Assays were conducted according to manufacturer’s protocol. Percent cell survival was analyzed and compared with mock infected wells.

### 2.6. Plaque Assay

Vero cells were seeded in 12-well plates at a density of 2 × 10^5^ cells per well. These were allowed to become confluent for 24 h. Eight ten-fold serial dilutions were conducted with each sample before being placed on the 12-well plates. Plates were incubated for 1 h and rocked every 15 min. Plates were overlayed with a 1:1 ratio of 1.0% agarose and Eagle’s Modified Essential Medium (without phenol red) supplemented with 5% FBS, 1% penicillin and streptomycin, 1% L-glutamine, 1% NEAA, and 1% sodium pyruvate. After 48 h, plates were fixed with 10% formaldehyde for 1 h. Plugs were removed and plates were stained with 1% crystal violet, 20% ethanol, and 79% diH_2_O. Plaque-forming units are represented as PFU/mL. Vero cells were seeded in 6-well plates at a density of 5 × 10^5^ cells per well and plugs were left for 72 h in the case of the Echovirus. Limit of detection for plaque assay is specified on VEEV TC-83 IC50 graph.

### 2.7. RNA Extraction and qRT-PCR Assay

Cells were lysed using TRIzol Reagent (ThermoFisher, 15596026, Waltham, MA, USA) and intracellular RNA was extracted using a Direct-zol Miniprep RNA kit (Zymo Research, R2052, Irvine, CA, USA) following manufacturer’s instructions and stored at −80 °C. Viral RNA was detected using Verso 1-Step RT-qPCR Mix (ThermoFisher, AB4101A, Waltham, MA, USA) on a One-Step Quantitative RT-PCR System using primer pairs (forward, CTGACCTGGAAACTGAGACTATG, and reverse, GGCGACTCTAACTCCCTTATTG) and probe (TACGAAGGGCAAGTCGCTGTTTACC) against the nsP1 region of the viral genome. A standard curve was generated using serial dilutions of VEEV TC-83 RNA at known concentrations. Absolute quantification was performed using StepOne software v2.3 based on the threshold cycle relative to the standard curve.

### 2.8. Negative-Strand RT-qPCR

Negative-strand synthesis was performed as previously described [23]. cDNA was generated using a specific primer to negative-strand RNA for VEEV TC-83, which contained a T7 promoter sequence attached at the 5′ end (T7-TC83-Neg 5′-GCGTAATACGACTCACTATATCCGTCAGCTCTCTCGCAGG-3′). A high-capacity cDNA reverse transcription kit (4368814, ThermoFisher, Waltham, MA, USA) was used to generate the negative-strand cDNA per the manufacturer’s instructions. For qPCR of negative-strand viral RNA, forward primer specific to the T7 promoter sequence (5′-GCGTAATACGACTCACTATA-3′) and reverse primer specific to VEEV TC-83 (5′-CAGGTACTAGGTTTATGCGC-3′) were utilized. qPCR for detection of viral negative strand used thermal cycling conditions adapted from PowerUp SYBR Green (A25742, ThermoFisher Scientific, Waltham, MA, USA) per the manufacturer’s instructions: 1 cycle at 50 °C for 2 min, 1 cycle at 95 °C for 2 min, 40 cycles at 95 °C for 15 s, 60 °C for 15 s, and 72 °C for 1 min using StepOnePlus™ Real-Time PCR system (ThermoFisher Scientific, Waltham, MA, USA). The ΔΔCt method was used to determine the fold change compared to the Mock average.

### 2.9. Western Blots

Cells were lysed in Blue Lysis Buffer composed of 25 mL 2× Novex Tris-Glycine Sample Loading Buffer SDS (Invitrogen LC2676, Waltham, MA, USA), 20 mL T-PER TissueProtein Extraction Reagent (ThermoFisher, 78510, Waltham, MA, USA), 200 µL 0.5 M EDTA pH 8.0, 3 complete Protease Cocktail tablets, 80 µL 0.1M Na3VO4, 400 µL 0.1 M NaF, and 1.3 mL 1M dithiothreitol. A total of 15 µL of cell lysate was separated by gel electrophoresis on a NuPAGE 4–12% Bis-Tris gel (Invitrogen, NP0322BOX, Waltham, MA, USA) and transferred to PVDF membrane (ThermoFisher, 88518, Waltham, MA, USA). The membrane was blocked in 1% BSA in TBS—0.1% Tween (TBST) solution (ThermoFisher, 37520, Waltham, MA, USA) for 30 min at room temperature. Primary antibody was incubated overnight at 4 °C in 1% BSA TBST. Rabbit anti-nsP1 antibody (ThermoFisher, MA-5-47057, Waltham, MA, USA) was used at 1:1000, goat and actin HRP (Abcam, ab49900, Cambridge, MA, USA) was used at 1:30,000. The membrane was washed three times for five minutes with TBST. Secondary antibody was prepared in 1% BSA TBST and incubated at room temperature for 1 h. Goat anti-rabbit HRP (Invitrogen, 32460, Waltham, MA, USA) was used at 1:1000. Membranes were washed once for five minutes with TBST and twice for five minutes with TBS. SuperSignal West Femto Maximum Sensitivity Substrate kit (ThermoFisher, 34095, Waltham, MA, USA) was used to image blots on a Chemidoc Imaging System (BioRad, 12003153, Hercules, CA, USA).

### 2.10. Statistical Analyses

Statistical analyses were conducted using unpaired, two-tailed *t*-tests in GraphPad Prism version 9.2.0 for Windows 10. Significance values are indicated using asterisks for * *p* < 0.05, ** *p* < 0.01, *** *p* < 0.001, and **** *p* < 0.0001; ns for not significant.

## 3. Results

### 3.1. Brilacidin Inhibits Alphavirus Replication in Vero Cells

**Figure 1:**
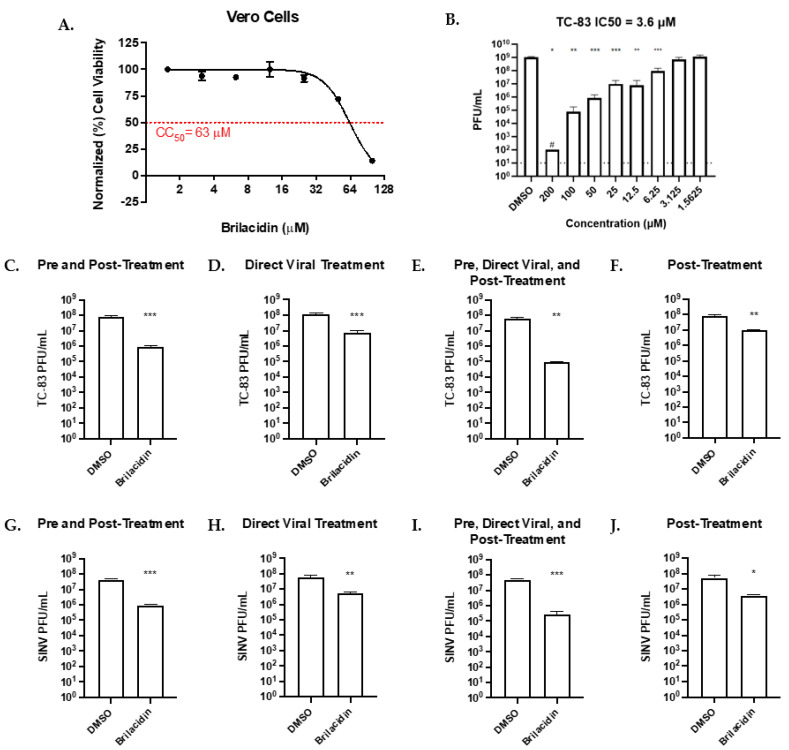
Brilacidin inhibits alphavirus replication in African green monkey kidney (Vero) cells. (**A**) Cytotoxicity of brilacidin was determined in Vero cells using CellTiter-Glo Luminescent Cell Viability Assay after 24 h of treatment. (**B**) Cells were pre-treated with brilacidin at varying concentrations along an 8-point curve. TC-83 was incubated for 1 h following the same concentration scheme. Pre-treatment was removed and cells were infected with TC-83. After 1 h, inoculum was removed and replaced with brilacidin-containing media. All samples were compared with DMSO control group. Supernatants were collected 16 h post-infection and viral titer was determined via plaque assay. A curve of inhibition was built and used to calculate inhibitory concentration 50% (IC50) of 3.6 µM. (**C**–**J**) Effects of various brilacidin treatments on VEEV TC-83 and SINV were determined. (**C**,**G**) Cells were pretreated with brilacidin (20 µM) for one hour and then infected with VEEV TC-83 or SINV (MOI of 0.1) for 1 h. After infection, brilacidin (20 µM)-containing media was applied. (**D**,**H**) VEEV TC-83 and SINV were directly treated with brilacidin at 20 µM for 1 h prior to infection. After infection, inoculum was removed, and media was applied. (**E**,**I**) Treatments described in (**C**,**G**) and (**D**,**H**) were combined. (**F**,**G**) Cells were infected with VEEV TC-83 or SINV (MOI of 0.1) for 1 h. After infection, the inoculum was removed and brilacidin (20 µM)-containing media was applied. All supernatants were collected at 18 hpi, and the viral titer was determined by plaque assay. Values are an average of 3 biological replicates ± standard deviation. # Indicates 1 biological replicate in IC50 data due to toxicity and samples falling below limit of detection (LOD). Dashed line indicates LOD. * *p* < 0.05, ** *p* < 0.01, *** *p* < 0.001.

The efficacy of brilacidin against VEEV-TC-83 and SINV was assessed in African green monkey kidney (Vero) cell. The toxicity of brilacidin was determined by incubating the cells with varying concentrations of brilacidin for 24 h. After the incubation period, the Cell Toxicity 50% (CC50) determined by Cell Titer-Glo assay was found to be 63 μM (Figure 1A). Under continuous brilacidin treatment condition, the IC50 value was determined in Vero cells (Figure 1B) to be 3.6 µM. Two replicates at 200 μM fell below the limit of detection (LOD) due to toxicity and as such this data point is based on one biological replicate.

Four treatment strategies were used to determine how brilacidin treatment may elicit an inhibitory outcome. (1) Pre-treatment and post-treatment were used to determine if early viral entry and post-entry steps, and viral RNA synthesis were inhibited. For pre-treatment and post-treatment, cells were first treated with brilacidin at 20 µM for one hour. Pre-treatment was then removed, and cells were infected at an MOI of 0.1 for one hour. Inoculum was removed and post-treatment of brilacidin at 20 µM was applied. (2) Direct viral treatment was used to determine if brilacidin can directly inhibit viral entry. Direct treatment was accomplished by independently treating the virus in media containing brilacidin for one hour prior to infection. (3) A combination of the pre-treatment, post-treatment, and direct viral treatments was used to determine if inhibition was the outcome of a combination of mechanisms that affected early entry, post-entry, and subsequent RNA synthesis steps. (4) Finally, post-treatment alone was also assessed to determine if brilacidin treatment-associated inhibition was independent of early entry and post-entry mechanisms. For post-treatment alone, cells were first infected at an MOI of 0.1 for one hour and after the viral inoculum was removed media containing brilacidin at 20 µM s was added. For each experiment/treatment strategy, a DMSO-treated control was included for comparison to brilacidin 20 µM. At 18 h post-infection (hpi), cell culture supernatants were collected and the viral titers were assessed via plaque assay, reporting an average of three replicates.

The pre-treatment and post-treatment strategy for VEEV TC-83 in Vero cells resulted in viral titers being reduced by 98.85% (*p* < 0.001, Figure 1C). Direct treatment of TC-83 alone led to a 93.91% reduction compared to the vehicle control (*p* < 0.001, Figure 1D). Combining these two treatment strategies further decreased the viral titer by 99.85% compared to vehicle control (*p* < 0.01, Figure 1E). Post-treatment alone also decreased the viral titer by 87.95% compared to the vehicle control (*p* < 0.01, Figure 1F). Similar results were seen with SINV following the four treatments used to assess the efficacy of brilacidin. Pre-treatment and post-treatment reduced viral titers by 97.85%, whereas direct treatment of SINV led to a 90.57% decrease compared to vehicle controls (*p* < 0.001, Figure 1G; *p* < 0.01, Figure 1H). Combining the pre-treatment, post-treatment, and direct viral treatment strategies reduced the viral titer by 99.43% compared to the vehicle control (*p* < 0.001, Figure 1I). Post-treatment alone also reduced SINV viral titer by 92.81% compared to the vehicle control (*p* < 0.05, Figure 1J). Overall, the data show that brilacidin significantly inhibits alphavirus replication by post-entry steps that impact positive and negative-strand RNA synthesis in infected cells.

**Figure 2:**
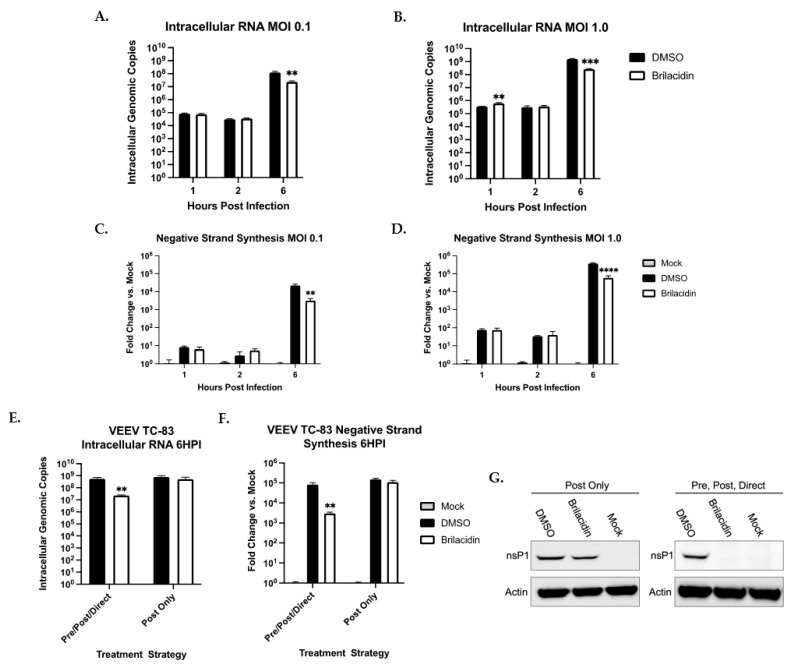
Brilacidin affects TC-83 viral replication post-entry. (**A**–**D**) Cells were pre-treated for 1 h with brilacidin (20 µM). TC-83 was incubated with brilacidin in media for 1 h. Pre-treatment was removed and cells were infected at an MOI of 0.1 or 1.0 for 1 h. Inoculum was removed and cells were post-treated for 2 and 6 h post-infection time points. The 1 h post-infection intracellular RNA were collected at time of inoculum removal. For 2 and 6 h time points, intracellular RNA were collected. RNA was extracted utilizing a Zymo Direct-zol RNA Miniprep Kit. (**A**,**B**) Positive-sense viral RNA was quantified via RT-qPCR with nsp1 primers. (**C**,**D**) Quantitative analysis of negative-sense RNA was completed via cDNA synthesis and qPCR. (**E**–**G**) Pre-/Post-/Direct treatment followed treatment described in (**A**–**D**). For post-treatment, cells were infected at an MOI of 0.1 for 1 h. Inoculum was removed and cells were treated with brilacidin (20 µM) until 6 h post-infection. (**E**) Positive-sense intracellular RNA was quantified using RT-qPCR. (**F**) Quantitative analysis of negative-sense RNA was completed via cDNA synthesis and qPCR. (**G**) Intracellular protein lysates were analyzed for nsp1 expression. Values are an average of 3 biological replicates ± standard deviation. ** *p* < 0.01, *** *p* < 0.001, **** *p* < 0.0001.

To further address if brilacidin treatment affected entry or early post-entry mechanisms in an infectious dose-dependent manner, independent quantification of intracellular RNA was performed at 1 h, 2 h, and 6 h post-infection at two different multiplicities of infection (MOI: 0.1 and MOI: 1.0) (Figure 2A,B). The quantification of incoming positive-stranded RNA at the 1 h and 2 h post-infection time points did not show differences from the DMSO-treated control suggesting that the treatment did not directly impact entry. However, a statistically significant reduction in positive-stranded RNA load was detected at 6 h post-infection, alluding to the inhibition of early post-entry steps that decreased positive-stranded RNA levels. This impact of the decrease in positive-stranded RNA levels at the 6 h time point was dependent on the pre-treatment step, as the post-treatment alone failed to achieve this outcome (Figure 2E). It can be reasoned that a decrease in the positive-stranded RNA levels will also result in a decrease in negative-stranded RNA synthesis, thus impacting viral RNA replication. Targeted inquiry of negative-stranded RNA levels by PCR supported this hypothesis as at the corresponding 6 h time point, negative-stranded RNA levels were lower in the brilacidin-treated samples as compared to the DMSO-treated controls in an infectious dose-dependent manner (Figure 2C,D). As we observed that the post-treatment alone did not result in viral inhibition as compared to the pre- and post-treatment, we conducted a direct comparison of the intracellular positive strand (Figure 2E) and negative strand (Figure 2F) under these specific conditions at the 6 h time point. The data agree with the plaque assay outcomes, indicating that the decrease in intracellular viral RNA occurred only in pre- and post-treatment conditions. We also reasoned that a decrease in intracellular viral positive RNA in the pre- and post-treatment condition will also result in the decreased production of the viral nonstructural proteins at the 6 h time point. We compared the amount of nsP1 under the two different treatment conditions by western blot analysis (Figure 2G), which also demonstrated that under pre- and post-treatment conditions, there was decreased nsP production. Thus, the inhibitory outcome of brilacidin was due to early post-entry events that impacted intracellular positive and negative-strand RNA levels, and by extension, also the amount of nonstructural viral proteins produced.

### 3.2. Brilacidin Inhibits Rift Valley Fever Virus Replication in HSAECs

**Figure 3:**
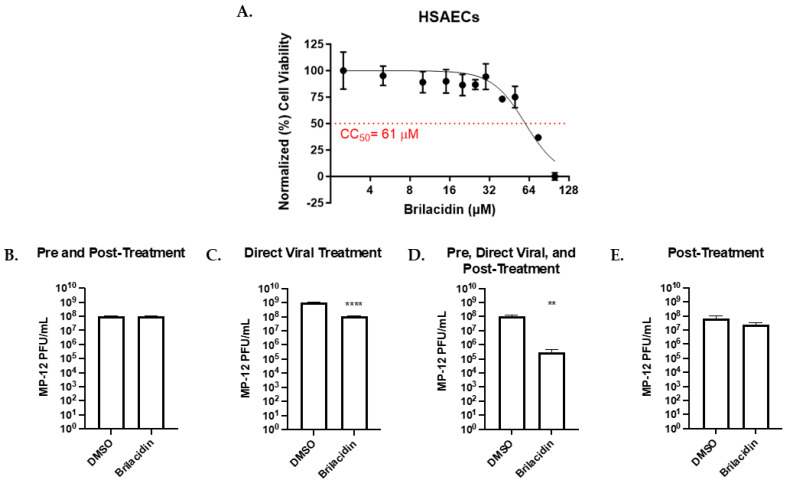
Brilacidin inhibits Rift Valley Fever virus (RVFV MP-12) replication in Human Small Airway Epithelial cells (HSAECs). (**A**) Cytotoxicity of brilacidin was determined in HSAEC cells using CellTiter-Glo Luminescent Cell Viability Assay after 24 h of treatment. (**B**–**E**) Effects of various brilacidin treatments on RVFV MP-12. (**B**) Cells were pre-treated with brilacidin (20 µM) for 1 h and then infected with MP-12 (MOI of 0.1) for 1 h. After infection, brilacidin (20 µM)-containing media was applied. (**C**) MP-12 was directly treated with brilacidin at 20 µM for 1 h prior to infection. After infection, inoculum was removed and media was applied. (**D**) Treatments described in (**B**,**C**) were combined. (**E**) Cells were infected with MP-12 (MOI of 0.1) for 1 h. After infection, the inoculum was removed and brilacidin (20 µM) containing media was applied. All supernatants were collected at 16 hpi, and the viral titer was determined by plaque assay. Values are an average of 3 biological replicates ± standard deviation. ** *p* < 0.01, **** *p* < 0.0001.

The potential for brilacidin to act as a broad-spectrum inhibitor of vector-transmitted, enveloped viruses was further assessed against RVFV. An initial toxicity screening for brilacidin in HSAECs was performed as described above. The CC50 of brilacidin was found to be 63 µM (Figure 3A). The treatment strategy used on alphaviruses was used to determine the efficacy of brilacidin on RVFV; the live-attenuated vaccine strain MP-12 was used in this set of experiments. A combination of pre-treatment and post-treatment with brilacidin at 20 µM did not yield a significant decrease in viral titer (Figure 3B) and post-treatment alone did not yield a decrease in viral titer (Figure 3E). Direct viral treatment saw a significant decrease in viral titer compared to the vehicle control with an 89.8% reduction in viral titer (*p* < 0.0001, Figure 3C). A combination of these two treatment strategies yielded an even further decrease to 99.7% (*p* < 0.01, Figure 3D). The proof of concept inhibition data presented for RVFV MP-12 alludes to a similar impact of brilacidin treatment on early post-entry mechanisms that contribute to viral inhibition.

### 3.3. Brilacidin Inhibits Virulent Alphaviruses and Bunyavirus

**Figure 4:**
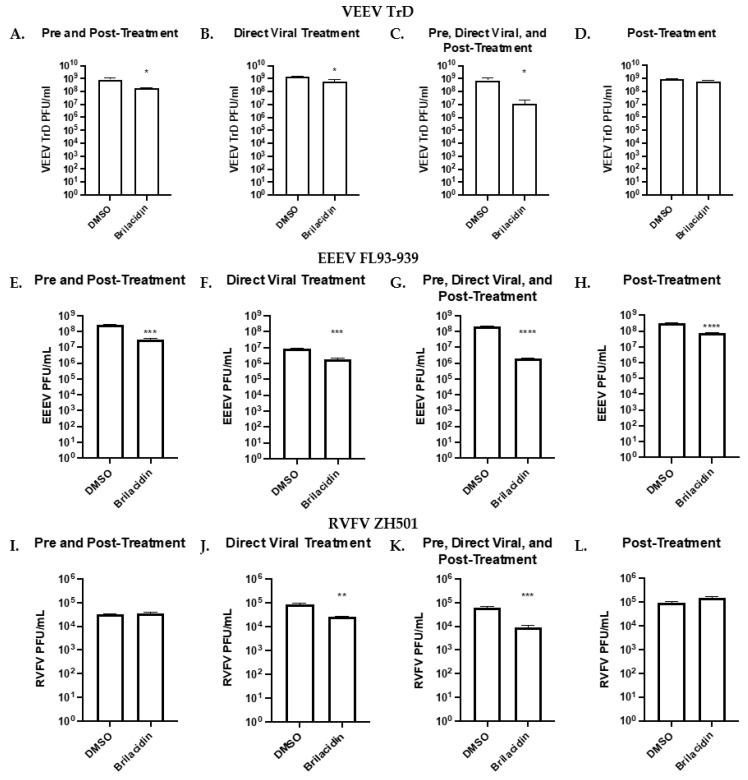
Brilacidin inhibits fully virulent alphaviruses and bunyavirus. (**A**–**L**) Effects of various brilacidin treatments on VEEV TrD, EEEV FL93-939, and RVFV ZH501. (**A**,**E**,**I**) Cells were pretreated with brilacidin (20 µM) for 1 h and then infected (MOI of 0.1) for 1 h. After infection, brilacidin (20 µM)-containing media was applied. (**B**,**F**,**J**) Viruses were directly treated with brilacidin at 20 µM for 1 h prior to infection. After infection, inoculum was removed, and media was applied. (**C**,**G**,**K**) Treatments described in (**A**,**B**) were combined. (**D**,**H**,**L**) Cells were infected (MOI of 0.1) for 1 h. After infection, the inoculum was removed and brilacidin (20 µM)-containing media was applied. VEEV and EEEV supernatants were collected at 18 hpi and RVFV was collected at 16 hpi. Viral titer was determined by plaque assay. Values are an average of 3 biological replicates ± standard deviation. * *p* < 0.05, ** *p* < 0.01, *** *p* < 0.001, **** *p* < 0.0001.

The effectiveness of brilacidin treatment against virulent strains of VEEV (VEEV TrD), EEEV (FL93-939), and RVFV (ZH501) was tested under BSL-3 conditions. Brilacidin treatment resulted in a decrease in viral titer when used against VEEV TrD following the same treatment strategy used with VEEV TC-83. Pre-treatment and post-treatment reduced viral titer by 77.84% (*p* < 0.05, Figure 4A), whereas direct treatment alone reduced viral titers by 57.98% (*p* < 0.05, Figure 4B) compared to the DMSO control. The combination of these treatments further reduced the viral titers by 98.35% (*p* < 0.05, Figure 4C), but post-treatment alone did not significantly impact viral replication and only decreased viral titer by 29.41% (Figure 4D). The effects of brilacidin on EEEV were more significant than VEEV TrD. Pre-treatment and post-treatment showed an 88.23% (*p* < 0.001. Figure 4E) decrease in viral titer, whereas direct treatment alone was 79.6% (*p* < 0.001, Figure 4F). Combining these two treatment strategies further reduced viral titer by 99.13% (*p* < 0.0001, Figure 4G) compared to the vehicle control. Unlike VEEV TrD, post-treatment alone did significantly reduce EEEV viral titer by 77.49% (*p* < 0.0001, Figure 4H).

These results further illustrate the potential of brilacidin to inhibit fully virulent alphaviruses and bunyavirus prototype pathogens.

### 3.4. Brilacidin Inhibits Echovirus Replication in Vero Cells

**Figure 5:**
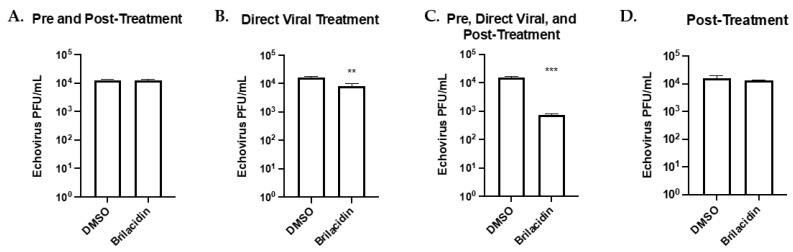
Brilacidin inhibits Echovirus replication in Vero cells. (**A**–**D**) Effects of various brilacidin treatments on Echovirus. (**A**) Cells were pretreated with brilacidin (20 µM) for 1 h and then infected (MOI of 0.1) for 1 h. After infection, brilacidin (20 µM)-containing media was applied. (**B**) Echovirus was directly treated with brilacidin at 20 µM for 1 h prior to infection. After infection, inoculum was removed, and media was applied. (**C**) Treatments described in (**A**,**B**) were combined. (**D**) Cells were infected (MOI of 0.1) for 1 h. After infection, the inoculum was removed and brilacidin (20 µM) containing media was applied. All supernatants were collected at 24 hpi, and the viral titer was determined by plaque assay. Values are an average of 3 biological replicates ± standard deviation. ** *p* < 0.01, *** *p* < 0.001.

An Echovirus was selected to determine if brilacidin was effective against a non-enveloped virus. Vero cells were used for all infections with this virus following the same treatments described in Figure 3. No significant difference in viral titer could be seen in pre-treatment and post-treatment as well as post-treatment alone (Figure 5A,D). Direct viral treatment yielded a 50.6% reduction in viral titer compared to the vehicle control (*p* < 0.01, Figure 5B). A combination of pre-treatment, post-treatment, and direct viral treatment resulted in an overall reduction of 95.2% (*p* < 0.001, Figure 5C).

### 3.5. Cell Viability during Infection and Treatment with Brilacidin

**Figure 6:**
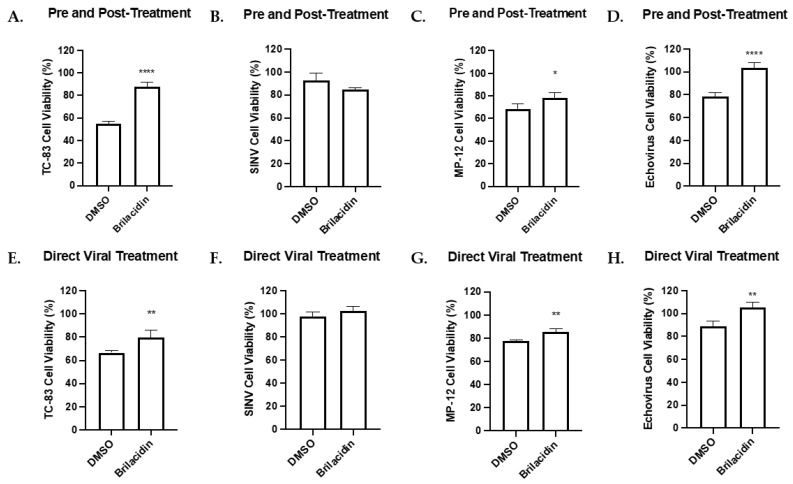
Brilacidin affects cell viability during infection. (**A**–**H**) Effects of pre- and post-treatment and direct viral brilacidin treatments on cell viability during infection with VEEV TC-83, SINV, RVFV MP-12, and Echovirus. (**A**,**B**,**E**,**F**) Vero cells, TC-83, and SINV were treated as described in Figure 1. After 18 hpi, cell viability was determined using CellTiter-Glo Luminescent Cell Viability Assay and compared to uninfected control. (**C**,**G**) HSAECs and MP-12 were treated as described in Figure 2. After 16 hpi, cell viability was determined using CellTiter-Glo Luminescent Cell Viability Assay and compared to uninfected control. (**D**,**H**) Vero cells and Echovirus were treated as described in Figure 4. After 24 hpi, cell viability was determined using CellTiter-Glo Luminescent Cell Viability Assay and compared to uninfected control. Values are an average of 4 biological replicates ± standard deviation. * *p* < 0.05, ** *p* < 0.01, **** *p* < 0.0001.

The effects of brilacidin on cell survival during infection were assessed for VEEV TC-83, SINV, RVFV MP-12, and Echovirus following the same previously used cell lines and treatments. An infected DMSO-treated control was included for comparison. Pre-treatment and post-treatment as well as direct treatment alone showed a significant increase (at least *p* < 0.05) in cell viability for VEEV TC-83, RVFV MP-12, and Echovirus, whereas no significant difference was seen for SINV (Figure 6A–H). The effects of combining pre-treatment, post-treatment, and direct viral treatment strategies or post-treatment alone showed a significant increase (at least *p* < 0.05) in cell viability for VEEV TC-83 (Supplementary Figure S2A,E), whereas no change in cell viability was seen for RVFV MP-12 (Supplementary Figure S2C,G). The effects of the treatment strategies on cells infected with SINV and Echovirus were mixed, with a decrease in cell viability seen in the combined treatment strategies for SINV and Echovirus and post-treatment for Echovirus (Supplementary Figure S2B,D,H) and no change in the survival of post-treated SINV-infected cells (Supplementary Figure S2F). These results generally show a modest increase in cell survival during infection with brilacidin treatment.

## 4. Discussion

The need for new antiviral therapeutic options is substantial due to a current lack of treatments and preventive vaccines with desirable safety profiles for the acutely infectious viruses discussed here. In this study, the alphaviruses VEEV, EEEV, and SINV, as well as the bunyavirus RVFV, were treated with brilacidin to assess the broad-spectrum antiviral potential of this defensin mimetic. Four treatment strategies were used to evaluate and further identify the impact of brilacidin on enveloped viruses and one non-enveloped virus. For the alphaviruses, VEEV TC-83, SINV, and EEEV, all four treatment strategies significantly inhibited viral replication. In the case of VEEV TrD, pre-treatment and post-treatment, direct viral treatment, and a combination of these treatments significantly reduced viral titers, whereas post-treatment alone had no significant effect. Additional viral genome quantification studies indicated that brilacidin treatment negatively affected both positive- and negative-strand RNA synthesis in the context of VEEV TC-83, thus alluding to early post-entry mechanisms being affected [18,24]. Direct treatment of RVFV with brilacidin resulted in viral inhibition indicating that there may be additional mechanistic differences between alphaviruses and bunyaviruses being inhibited by brilacidin. This is not surprising because alphaviruses are positive-stranded viruses that need to be converted to negative strands for RNA replication, whereas the bunyavirus genome is negative stranded. The engagement of the secretory pathway for viral egress is also fundamentally different between these two pathogens which may also contribute to late events being impacted, which were not investigated as part of this work. The non-enveloped Echovirus was also only inhibited by brilacidin when applied as a direct treatment although not to the same extent as seen with most of the enveloped viruses thus further underscoring the impact of brilacidin on enveloped viruses specifically. Interestingly, previously published studies with adenovirus and host defensins showed similar inhibitory events as observed in the current study, pointing towards a mechanism that involves intracellular viral RNA [25,26]. Our studies with the host defense peptide LL37 elicited reasonably comparable inhibition of VEEV TC-83 and TrD as observed with this defensin mimetic, following a pre- and post-infection treatment strategy [27] similar to the one followed in the studies included in this manuscript. Similar mechanisms of inhibition observations were made when brilacidin was compared to LL16, another defensin [28].

Brilacidin has previously been tested in over 500 human subjects across multiple clinical indications where it has been shown to be efficacious and well-tolerated [29,30,31,32,33]. Brilacidin, acting in synergy with other antiviral AMPs or antiviral drugs, may help arrest multiple parts of the viral lifecycle, resulting in better disease outcomes and a reduced risk for antiviral resistance. Deploying brilacidin as part of a prophylactic strategy to prevent early post-entry mechanisms and inhibit the intracellular production of new viral RNA could further prevent severe inflammatory effects observed during later stages of viral infection. Additionally, other enveloped viruses should be tested to further inform brilacidin’s broad spectrum potential and mechanism of action. Although brilacidin holds translational promise as a broadly effective countermeasure, there are additional studies to be performed in the context of the viruses included in this manuscript including in vitro specificity studies, expanded mechanism of action studies that focus on intracellular events including vesicular interactions, and impact on the innate immune inflammatory events, and in vivo dosing assessments that are pertinent to acute viral infections before the compound can become mainstream as a broad spectrum intervention strategy.

## Data Availability

Data are contained within the article or Supplementary Material.

## Supplementary Materials

The following supporting information can be downloaded at: https://www.mdpi.com/article/10.3390/microorganisms12010054/s1, Figure S1: IC50 analysis of MP-12 in Vero cell line. Figure S2: Brilacidin affects cell viability during infection.
