# Supplementary material for: Brilacidin as a Broad-Spectrum Inhibitor of Enveloped, Acutely Infectious Viruses
Source: Microorganisms. 2023 Dec 28;12(1):54. doi: 10.3390/microorganisms12010054 (PMC10819233; doi:10.3390/microorganisms12010054)
Supplement: Supplementary file 1 [file microorganisms-12-00054-s001.zip › microorganisms-2665520-supplementary.pdf]

A.

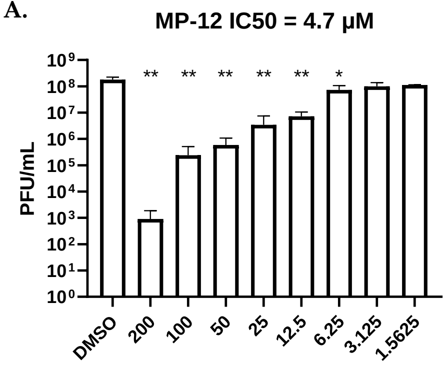

Supplemental Figure S1. Brilacidin affects MP-12 replication in Vero cells at varying concentrations. (A) Cells were pre-treated with brilacidin at varying concentrations along an 8 point curve. MP-12 was incubated for 1 hour following the same concentration scheme. Pre-treatment was removed and cells were infected with MP-12. After 1 hour, inoculum was removed and replaced with brilacidin containing media. All samples were compared with DMSO control group. Supernatants were collected 16 hours post-infection and viral titer was determined via plaque assay. A curve of inhibition was built and used to calculate inhibitor concentration 50% (IC50) of 4.7 µM. * $p < 0.05$, ** $p < 0.01$.

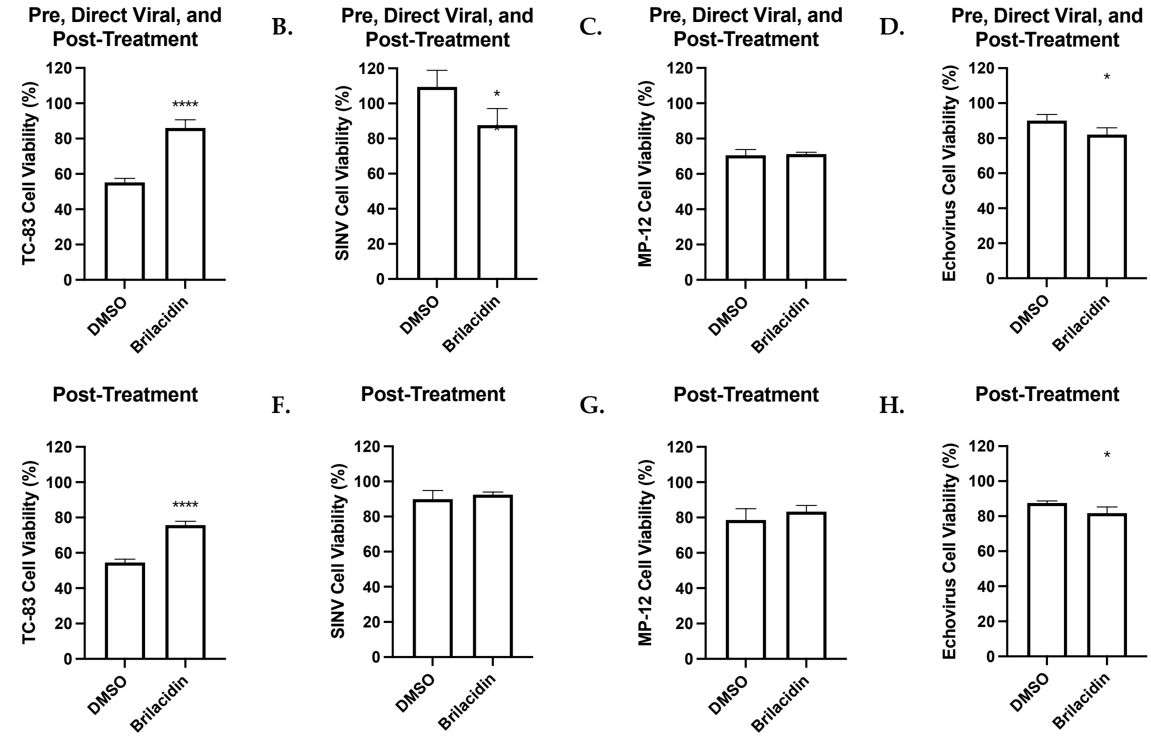

Supplemental Figure S2. Brilacidin affects cell viability during infection. (**A-H**) Effects of pre and post-treatment and direct viral brilacidin treatments on cell viability during infection with VEEV TC-83, SINV, RVFV MP-12 and Echovirus. (**A-B, E-F**) Vero cells, TC-83, and SINV were treated as described in Figure 1. After 18hpi cell viability was determined using CellTiter-Glo Luminescent Cell Viability Assay and compared to uninfected control. (**C, G**) HSAECs and MP-12 were treated as described in Figure 2. After 16hpi cell viability was determined using CellTiter-Glo Luminescent Cell Viability Assay and compared to uninfected control. (**D, H**) Vero cells and Echovirus were treated as described in Figure 4. After 24hpi cell viability was determined using

CellTiter-Glo Luminescent Cell Viability Assay and compared to uninfected control. Values are an average of 4 biological replicates ± standard deviation. * $p < 0.05$, **** $p < 0.0001$.
